# Knowledge and practice of iodine salt utilization and associated factors among pregnant women attending antenatal care in public health facilities in Addis Ababa, Ethiopia

**DOI:** 10.3389/fnut.2025.1529842

**Published:** 2025-06-19

**Authors:** Ejigu Girma, Habtamu Molla Ayele, Elzabeth Seyoum, Getachew Weldeyohannes

**Affiliations:** ^1^Department of Maternal and Child Health, Yeka Sub City Health Office, Addis Ababa, Ethiopia; ^2^Maternal and Child Health Directorate, Federal Ministry of Health, Addis Ababa, Ethiopia; ^3^Department of Public Health, Yekatit 12 Hospital Medical Colleges, Addis Ababa, Ethiopia

**Keywords:** iodized salt, utilization, knowledge, practices, pregnant women, Addis Ababa

## Abstract

**Background:**

Globally, iodized salt deficiency affects more than 2.2 billion people, and the effect is severe in pregnant women. Several factors contribute to the insufficient utilization of iodized salt in the population. Knowledge and practice of iodized salt utilization contribute to improving the utilization of iodized salt among pregnant mothers.

**Objective:**

This study aimed to assess the knowledge and practice of iodized salt utilization and its associated factors among pregnant women attending antenatal care at public health facilities in Addis Ababa, Ethiopia.

**Method:**

A facility-based cross-sectional study was conducted in the primary public health centers of selected sub-cities in Addis Ababa from 6 May to 20 July 2022, using interviewer-administered structured questionnaires. The total sample size was 472 participants. Data entry was performed using Epi Data version 3.1 and imported to SPSS version 23 to perform various analyses. Bivariable logistic regression analysis was performed to identify candidate variables with a *p-*value <0.25, and then multinomial logistic regression analysis was conducted to determine the level and factors associated with iodized salt utilization. In the multivariable model, adjusted odds ratios, together with their corresponding 95% CI, were calculated to assess the strength of association and to declare statistical significance at a *p*-value <0.05.

**Results:**

Among the 463 respondents, 190 (41.1%) had good knowledge of iodized salt, whereas 270 (58.3%) had good practices for iodized salt utilization. Occupation, average monthly income, and the number of pregnancies were significantly associated with knowledge of iodized salt utilization. Educational level, average monthly income, and the number of ANC visits were significantly associated with good practices for iodized salt utilization.

**Conclusion and recommendations:**

Based on the findings of this study, it can be concluded that women attending antenatal care have low levels of iodized salt knowledge and practice in the study area. Nutritional interventions, such as nutrition education, should be provided to the community, particularly for pregnant mothers.

## Introduction

All human beings require a balanced amount of nutrients for the proper functioning of the body system. Nutrition is a fundamental pillar of human life, health, and development throughout the entire lifespan (1). Approximately 40 different nutrients are essential for health. If any of these are deficient in diet, the person will not be fully healthy and will not be able to resist the agents of the disease (2). Micronutrient deficiencies (hidden hunger), particularly zinc, iron, iodine, and vitamin A deficiencies, continue to inflict substantial global health, economic, and social encumbrances. Humans require iodine for various purposes, including psychological, metabolic, and physiological functions. Iodine is an essential micronutrient required for growth and development (3). Iodine is essential for the production of thyroid hormones that are vital for proper brain development and function.

Iodine deficiency (ID) is a major public health problem affecting populations worldwide, predominantly pregnant women and young children (4). It is one of the most important causes of preventable mental impairment worldwide (5). Iodine deficiency causes inadequate thyroid hormone production and has many adverse effects on growth and development (6). Many of these adverse consequences are collectively referred to as iodine deficiency disorders (IDDs) (7). Poor iodine intake is a major public health problem in women of reproductive age, and lactating women are particularly susceptible to iodine deficiencies, which expose them to irreversible, mentally impaired babies. It is also documented that it causes abortions, stillbirths, congenital abnormalities, cretinism, dwarfism, goiter and impaired mental function, muscular dystrophy, spontaneous miscarriages, squinting, hypothyroidism, and stunting (7, 8). The most significant consequence of inadequate iodine intake, particularly during fetal development and early childhood, is impaired brain development and function, which can lead to severe intellectual disability and cretinism (9). IDDs are a major public health problem worldwide, particularly in developing countries (10). Approximately two billion people (33.3%) of the world's population live in areas with iodine deficiency and are at risk of complications (7, 11), of which at least 321 million Africans are at risk of iodine deficiency (12). The coverage of iodized salt varies by region, ranging from 90% in Asia and the Pacific to 40%−60% in sub-Saharan Africa. Utilization rates differ significantly between countries, ranging from <10% to 90%. Sudan, Mauritania, Guinea-Bissau, and Gambia have utilization rates below 10%, whereas Burundi, Kenya, Nigeria, Tunisia, Uganda, and Zimbabwe have reached the Universal Salt Iodization (USI) target (7).

In Ethiopia, iodine deficiency threatens 35 million people, contributing to 50,000 prenatal deaths annually (7). Some pocket areas in the country have a Target Goiter Rate (TGR) of 50%−95%, and it is estimated that 100,000 children born each year are at risk of intelligence quotient (IQ) impairment due to ID, resulting in an average loss of 22 million dollars in productivity each year (13). To address this issue, universal salt iodization is considered the most efficient, safe, and sustainable approach for eliminating iodine deficiency disorders (14). Encouragingly, 76% of households worldwide consume adequate amounts of iodized salt (15). According to the WHO, the optimal level of iodized salt utilization for preventing IDDs is achieved when the salt contains 15–40 parts per million (ppm) of iodine, and for households, access to iodine salt should be 90% and above (16).

During pregnancy, women experience a heightened demand for energy and nutrients to support the developing fetus and maternal tissues involved in pregnancy. Achieving a proper dietary balance is crucial to ensure that the fetus receives adequate energy for growth without depleting the mother's own tissue reserves to sustain pregnancy (17). The inadequate health and nutrition of women, along with insufficient care, contribute to death during pregnancy and childbirth, which in turn affects the wellbeing and survival of infants and children (18).

It is essential for pregnant and breastfeeding women to increase their intake of calories, protein, calcium, folic acid, iodine, and iron. Adolescents, underweight women, obese women, those with ongoing nutritional issues, smokers, individuals who consume alcohol or drugs, low-income women, and those with chronic conditions such as diabetes or anemia are particularly vulnerable to nutritional deficiencies during pregnancy (19). Pregnancy greatly influences the thyroid gland and thyroid function. There is a 50% increase in the production of thyroid hormones, such as thyroxine (T4) and triiodothyronine (T3), during pregnancy due to physiological changes (20). The maternal iodine requirement to maintain maternal euthyroidism and the need for iodine are required by the fetus, and increased renal excretion of iodine during pregnancy causes pregnant women to fall under a highly vulnerable group for iodine deficiency (21). Although government health sector development programs exist, poor iodine intake in women continues to be a serious problem in Ethiopia (22). Iodized salt can lose its iodine content during handling and cooking because of gaps in knowledge regarding its proper use. In households, factors affecting inadequate intake of iodized salt are age, sex, residence, occupational status, educational status, religion, salt containers, knowledge regarding iodized salt availability, duration of storage, and checking the status of salt while purchasing (8).

Antenatal care services are an opportunity for pregnant women to provide information on the use of iodine salt and its methods of utilization in Ethiopia. This study aimed to assess the knowledge and practice of iodized salt utilization and its associated factors among pregnant women attending the Antenatal Care (ANC) outpatient department (OPD) at public health facilities in Addis Ababa, Ethiopia.

## Methods and materials

### Study area and period

The study was conducted among ANC attendants at selected health centers of the Addis Ababa City Administration. Addis Ababa, the largest city of Ethiopia, has a population of 3.24 million with an annual growth rate of 3.8%, is located at 901′48″ N and 38044′24″ E, 2355 m above sea level, and landed at 527 km^2^. In Addis Ababa City Administration, there are 11 sub-cities and 117 district-level administrative offices. Yeka, Nefassilk Lafto, and Lemi Kura are among the 11 sub-cities of Addis Ababa (23).

According to the 2022 district-based plan report from the Addis Ababa Health Bureau, the total population of Nefassilk Lafto sub-city was estimated to be 376,880, with a male population of 184,671 and a female population of 192,209. Among them, 130,551 were women of reproductive age, and the annual number of pregnant women was 8,781. The second sub-city was Lemi Kura, which had a total estimated population of 344,944, with a male population of 169,023 and a female population of 175,092. Among them, 119,489 were women of reproductive age, and the annual number of pregnant women was 8,037. The third sub-city was Yeka sub-city, which had a total population of 355,473; the male population was 174,182 and the female population was 181,291. Among these, 123,136 were women of reproductive age, and the annual number of pregnant women was 8,282 (23).

The study was conducted from 6 May to 20 July 2022, in selected sub-cities in Addis Ababa, Ethiopia.

### Study design

This study used a descriptive cross-sectional study design.

### Source population

The source population for the study was all pregnant women attending ANC clinics in public primary healthcare facilities in Addis Ababa.

### Study population

The study population consisted of randomly selected pregnant women who fulfilled the inclusion criteria and attended the ANC clinics of the selected public primary healthcare facilities in Addis Ababa.

### Sample size determination

Sample size estimation was performed for each specific objective using the single and double population proportion formula, and the largest sample size of the specific objectives was considered as the total sample size of this study. Accordingly, the following assumptions were made to determine the sample size:

**Objective 1:** To determine knowledge of iodized salt utilization among pregnant women visiting ANC clinics.

The sample size was calculated using a single population proportion formula, taking the proportion of nutritional knowledge, attitudes, and practices among pregnant women who attended antenatal care at public hospitals in Addis Ababa, Ethiopia, as 27% (17). In addition, a 5% marginal error (d), confidence interval of 95%, and non-response rate of 0.5% were used.

Based on these assumptions, the sample size was calculated as follows.


$$\begin{array}{l} n=\frac{z_{1-\alpha /2}^{2}p\left(1-p\right)}{d^{2}} \end{array}$$

Where:

n = Sample size.

α = Level of significance (set at 0.05).

z = the standard normal deviate with 95% CI (1.96).

p = the proportion of nutritional knowledge, attitudes, and practices among pregnant women who attend antenatal care from the previous study = 27%.

d = Degree of precision 5%.


$$\begin{array}{l} n=\frac{1.96^{2}\;\times \;0.27\left(1–0.27\right)}{{\left(0.05\right)}^{2}}=300 \end{array}$$

The total sample size was set to 300. Therefore, with adjustment for the non-response rate of 1.05% and a design effect of 1.5, the final sample size was 472 study participants.

**Objective 2:** To determine the practice of iodized salt utilization among pregnant women who visit ANC clinics.

The sample size was calculated using a single population proportion formula, with the proportion of utilized iodine as 25% from studies conducted in the Mecha district of Northwest Ethiopia (24).

Based on these assumptions, the sample size was calculated as follows.


$$\begin{array}{l} n=\frac{z_{1-\alpha /2}^{2}p\left(1-p\right)}{d^{2}} \end{array}$$

Where:

n = Sample size.

α = Level of significance (set at 0.05).

z = the standard normal deviate with 95% CI (1.96).

p = the proportion of iodized salt utilization from the previous study = 25%.

d = Degree of precision 5%.


$$\begin{array}{l} n=\frac{1.96^{2}\;\times \;0.25\left(1–0.25\right)}{{\left(0.05\right)}^{2}}=288 \end{array}$$

The total sample size in this study was 288. Therefore, with adjustment for the non-response rate of 1.05% of the total sample size and a design effect of 1.5, the final sample size was 453 study participants.

**Objective 3:** Determination of sample size for the third specific objective.

The sample size for the third objective was calculated using the double-population proportion formula with the following assumptions: a 95% confidence interval, 80% power, a ratio of exposed to non-exposed as 1:01, and by taking the odds ratio and percentage of outcome in the unexposed group from previous studies. By substituting the above assumptions into the Epi Info version 7 software stat calc programs, they are summarized in Table 1.

**Table 1:** Sample size calculation for factors associated with iodine utilization among pregnant women attending ANC.

**Factors (variables)**	**CI %**	**Power %**	**OR**	**Ratio (Unexposed: Exposed)**	**% outcome in unexposed group**	**% outcome in exposed group**	**Final sample size**	**References**
Formal education	95	80	0.44	1:01	58.27	38	208	(25)
Occupation	95	80	0.032	1:01	91.89	26.9	22	(25)
Income	95	80	3.08	1:01	27.7	54.166	22	(25)

As indicated in the Table 1, the sample size calculated for the first objective for knowledge of iodine utilization among pregnant women attending ANC was greater than the sample size calculated for the second objective for practice regarding iodized salt utilization among pregnant women who visited ANC clinics and factors associated with iodine utilization among pregnant women attending ANC. Therefore, the sample size calculated for the first objective, which was 472, was used for the present study.

### Sampling technique

Multistage sampling was performed in this study. The Yeka, Nefassilk Lafto, and Lemi Kura sub-cities were selected using simple random sampling as the primary sampling unit. Since 30% of the sub-cities were considered, it may provide a better representation of Addis Ababa City Administration health facility ANC-attendant mothers.

Once the sub-cities were selected by simple random sampling, three health centers were randomly selected from each sub-city. Nefassilk Lafto sub-city has eight governmental health centers (HCs). From these eight HCs, Woreda one HC, Woreda two HC, and Woreda 11 HC were randomly selected. Lemi Kura sub-city has 10 governmental health centers. From these 10 HCs, Woreda three HC, Woreda six HC, and Woreda 10 HCs were selected. The Yeka sub-city has 10 governmental health centers. From those 11 HCs, Kotebe HC, Yeka HC, and Yeka Woreda, 12 HCs were randomly selected (23).

**Figure 1:**
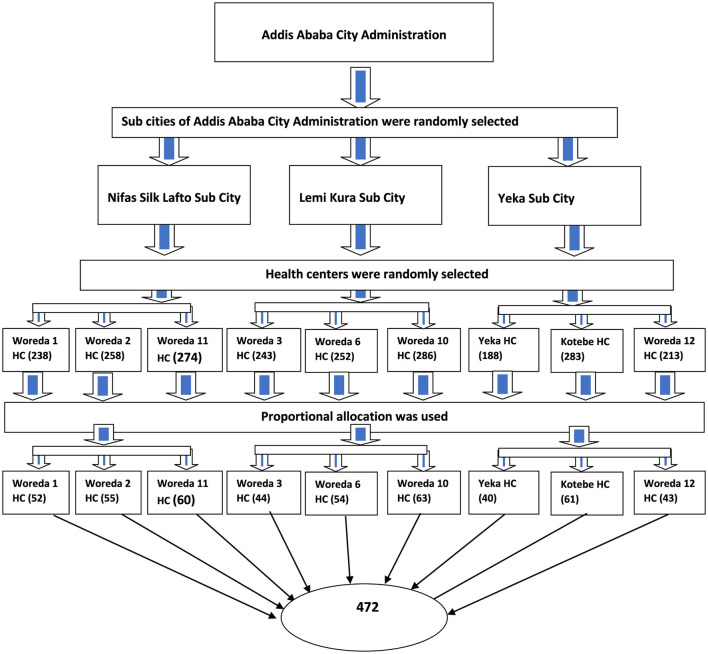
Schematic presentation of sampling procedure on iodized salt utilization and associated factors in Addis Ababa, Ethiopia.

Finally, nine health centers were selected as study units, and the total sample size was proportionally allocated to each woreda or district. The list of ANC attendants from the selected health centers was used as a sampling frame to select participants for the study using a simple random sampling technique from each health center, as shown in Figure 1.

### Inclusion and exclusion criteria

#### Inclusion criteria

- The inclusion criteria were pregnant women who attended ANC clinics at selected health centers.

#### Exclusion criteria

- Pregnant women with mental disorders and other patients who were in critical condition or could not talk were excluded from the study.
- Patients with salt restrictions (chronic hypertension) were excluded.

### Data collection procedure

Data were collected through face-to-face interviews during client visits to the ANC OPD at the exit of the ANC service using a structured questionnaire from 6 May to 20 July 2022. A structured tool, validated through a pilot study adapted from different literature (3, 4, 7, 17, 24–26), was prepared in English, translated into the local language of Amharic, and then translated back into English to check for consistency.

The questionnaire comprises three basic parts: the first part is about the background characteristics of the client, including the demographic and socioeconomic characteristics; the second part includes iodized salt knowledge; and the third part is the utilization or practice-related questions. For data collection, five BSc nurses or equivalent educational background holders and one supervisor who was not working in the selected health facilities were recruited for data collection.

### Study variables

#### Dependent variables

- Maternal knowledge of iodized salt utilization.
- Maternal practice of iodized salt utilization.

#### Independent variables

Demographic and socioeconomic factors such as age, sex, occupation, education level, religion, family income, ever heard of iodized salt, lists of places of hearing, respondents who had heard of the importance of iodized salt consumption during pregnancy, storage of iodized salt at home, place of storage, and the right time to add iodized salt to meals.

### Operational definition

#### Storage of iodized salt

Mothers who store the purchased iodized salt for more than 2 months were considered to have a longer storage time, and those who stored for <2 months were considered to have a shorter storage time (13).

- Good knowledge: The highest knowledge of iodized salt score was 21, and the average score was 10.5. Good knowledge was considered when the participant responded correctly to more than 50% of the knowledge questions (26).
- Poor knowledge: When the participant answered < 50% (the average score) of the knowledge questions (26).
- Good practice: The highest utilization of iodized salt score was 9 and the average score was 4.5. The participant had good practice when more than 50% of the practice questions were answered correctly (26).
- Poor practice: When the participant answered < 50% (4.5 questions) of the knowledge questions (26).

### Data analysis procedure

Data were cleaned and entered into Epi Data version 3.1 application and imported into SPSS version 23, a statistical package used for Windows analysis. Descriptive analyses, such as proportions, percentages, frequency distribution, and measures of central tendency, were used to present the information. Descriptive analyses were used to report frequencies and proportions to describe the characteristics of the study population for categorical variables, such as gender, language, education, and marital status. The knowledge and practice of iodized salt among participants were measured based on whether the participant answered < 50% of the knowledge and practice questions. Furthermore, it was defined as good or poor.

Bivariate logistic regression analysis was performed with odds ratios (ORs) and 95% confidence intervals (CIs), and Hosmer and Leme showed goodness of fit. It was performed to identify candidate variables for iodized salt utilization at a *p*-value of < 0.25, and then multinominal logistic regression analysis was conducted to determine the knowledge, practice, and factors associated with iodized salt utilization. In the multivariable model, adjusted odds ratios, together with their corresponding 95% CI, were calculated to control for the influence of potential confounding variables, assess the strength of association, and declare statistical significance at a *p-*value of < 0.05. Thus, the independent effect of each explanatory variable on the dependent variable was determined while controlling for the others.

### Data quality management

All questionnaires were checked daily by a supervisor for completeness, and the principal investigator monitored the overall quality of data collection. The collected data were stored in the form of a file in a secure place, where no one accessed it, except the investigator. In addition, the investigators ensured careful data entry and thoroughly cleaned the data before the commencement of analysis.

The principal researcher trained all data collectors and supervisors on the study objectives, purpose, and interview techniques based on the research instrument. During the data collection period, a daily debriefing was conducted to ensure that the target objective for the day was achieved. Cronbach's alpha was used to determine the reliability of questionnaire items.

## Results

### Sociodemographic characteristics of respondents

A total of 463 women visiting a health facility for ANC services were interviewed, with a 98% response rate. Two hundred and three (43.8%) respondents were aged between 35 and 44 years. The mean and standard deviation (SD) of the respondents age were 28.23 years and 5.84 years, respectively. More than half of the respondents, 257 (55.8%), were Orthodox religious followers, 290 (47.3%) were housewives, and 403 (87%) were married. Regarding the respondents' educational level, 154 (33.3%) attended diplomas and above. On the other hand, more than two-thirds, 323 (69.8%), of participants lived in the respondents' homes. The monthly income of 288 (63.1%) respondents was more than 1,500 ETB (Table 2).

**Table 2:** Sociodemographic characteristics of study participants in Addis Ababa city, Ethiopia, 2022 (*n* = 463).

**Variables**	**Possible options**	**Frequency (n)**	**Percentage (%)**
Health center (Nefassilk Lafto SC)	Woreda 1 HC	59	12.8
	Woreda 2 HC	52	11.2
	Woreda 11 HC	53	11.4
Health center (Lemi Kura SC)	Woreda 3 HC	42	9.1
	Woreda 6 HC	53	11.4
	Woreda 10 HC	62	13.4
Health center (Yeka SC)	Yeka HC	40	8.6
	Kotebe HC	61	13.2
	Woreda 12 HC	41	8.9
Women's age	15–24	22	4.8
	25–34	165	35.6
	35–44	203	43.8
	45–49	73	15.8
Religion	Orthodox	257	55.5
	Protestant	73	15.8
	Muslim	110	23.8
	Catholic	19	4.1
	Other Specify	4	0.9
Occupation	Government	112	24.2
	Non-Government	73	15.8
	Self employed	59	12.7
	House Wife	219	47.3
Marital status	Marred	403	87
	Single	38	8.2
	Divorced	22	4.8
Educational level	No formal education	66	14.3
	Primary education	131	28.3
	Secondary education	112	24.2
	Diploma and above	154	33.3
Number of people lived in respondents' home	Two	13	2.8
	Three	127	27.4
	Four and above	323	69.8
Monthly income (ETB)	< 500	25	5.4
	500–1,500	60	13
	More than 1500	288	62.2
	Not known	90	19.4

### Reproductive and ANC follow-up related characteristics of study participants

One hundred seventy-six (38%) participants had at least one pregnancy before. Approximately 155 (33.5%) respondents had a history of stillbirth or abortion. Regarding the reason for stillbirths or abortions, 201 (43.4%) participants did not know the reason. More than half of the women, 273 (59%), visited the health facility for ANC. More than half of the participants (52.5%) responded that their counselors were general practitioners.

The majority of participants, 401 (86.6%), received information during ANC follow-up. Most participants received information through counseling on hygiene and sanitation, nutrition, danger signs, and birth preparedness. The majority of participants (76.7%) said that no information was provided regarding iodine in ANC counseling (Table 3).

**Table 3:** Reproductive and ANC follow-up related characteristics of the study participants of Addis Ababa city, Ethiopia, 2022 (*n* = 463).

**Variables**	**Possible options**	**Frequency (n)**	**Percentage (%)**
Number of pregnancies	One	176	38
	Two	127	27.4
	Three	99	21.4
	Four and above	61	13.2
Stillbirth or abortion	No	308	66.5
	Yes	155	33.5
The reason of stillbirth or abortion	Rh in compatibility	30	6.5
	Accident	36	7.8
	Fetal distress	69	14.9
	Bleeding	65	14
	Nutrition	62	13.4
	I don't know	201	43.4
Number of ANC visit	One	32	6.9
	two to three	158	34.1
	Greater than or equal to four	273	59
ANC counselor	HO or BSc	211	45.6
	GP	243	52.5
	Midwife	9	1.9
Received health education	No	62	13.4
	Yes	401	86.6
Reported information provided during ANC follow-up	Hygiene and sanitation	24	5.2
	Nutrition	17	3.7
	Danger sign	33	7.1
	Birth preparedness	31	6.7
	All the above	358	77.3
Reported information provided about iodine	No	355	76.7
	Yes	108	23.3

### Knowledge of pregnant women on iodine and iodized salt utilization

The majority of the participants, 301 (65%), reported that they had heard about iodine. However, 357 (77.1%) and 283 (61.1%) of the participants had awareness of iodine deficiency disorder (IDD) and its effects on health, respectively. More than half (57%) of the participants were aware of the existence of iodine deficiency in Ethiopia. In general, the overall responses given by the respondents regarding their knowledge of iodized salt utilization revealed that 190 (41.1%) of the respondents were found to be in good knowledge about iodized salt utilization, while 273 (58.9%) had poor knowledge about iodized salt utilization (Table 4).

**Table 4:** Knowledge characteristic of pregnant women about iodine and iodized salt utilization in selected health centers of Addis Ababa, Ethiopia, 2022.

**Variables**	**Category**	**Frequency (*n =* 463)**	**Percentage (%)**
Heard about iodine micronutrient	Yes	301	65
	No	162	35
Awareness of iodine deficiency problems in the environment and insufficient intake of iodine in the human body	Yes	357	77.1
	No	106	22.9
Effect of iodine deficiency on the human body	Yes	283	61.1
	No	180	38.9
Existence of iodine deficiency problems in Ethiopia	Yes	264	57
	No	199	43
Method of prevention of IDDS	Using iodized salt	34	7.3
	Physical activity	223	48.2
	Making TATO	30	6.5
	Other	36	7.8
	I don't know	140	30.2
Heard about iodized salt	Yes	367	79.3
	No	96	20.7
Types of salt available in the market	Regular	19	4.1
	Iodized	18	3.9
	Mixed	426	92
Knowledge about every salt containing iodine	Yes	174	37.6
	No	289	62.4
Knowledge about the importance of using iodized salt	Yes	346	74.7
	No	117	25.3
Knowledge about important sources of iodine	Fish	157	33.9
	Meat	13	2.8
	Vegetable oil	11	2.4
	Dairy product	17	3.7
	Do not know	265	57.2
Advantage of iodized salt over non-iodized salt	Better test	55	11.9
	Easily soluble	112	24.2
	Replace iodine	169	36.5
	Do not know	127	27.4
Knowledge about lack of iodine could cause pregnant women to give birth before EDD	Yes	80	17.3
	No	108	23.3
	Do not know	275	59.4
Knowledge about iodine deficiency will make children learn below-normal	Yes	103	22.2
	No	102	22
	Do not know	258	55.7
Family suffered from goiter with cooked iodized salt	Yes	58	12.5
	No	208	44.9
	Do not know	197	42.5
Can differentiate iodized and non-iodized by looking	Yes	134	28.9
	No	143	30.9
	Do not know	186	40.2
Reason for buying iodized salt	I know it is healthy	218	47.1
	Other salts are not available	135	29.2
	Do not know	110	23.8
Knowledge about salt packing has information about contents	Yes	282	60.9
	No	106	22.9
	Do not know	75	16.2
Overall knowledge of pregnant mother	Poor knowledge	273	58.9
	Good knowledge	190	41.1

### Practice of utilization of iodized salt

Approximately 399 (86.2%) women bought and used mixed salt (iodized and non-iodized). Of the total respondents, 43% knew about the right timing for adding iodized salt to meals.

Most participants, 409 (88.3%), mentioned that the salt they were consuming in their homes was usually purchased from mini-shops, even though about 8.6% and 3% of the participants purchased it from customer associations and open markets, respectively. Approximately 384 participants (82.3%) used containers with lids to store salt at home, and 413 (89.2%) were exposed to sunlight. More than half of the participants (51.2%) kept iodized salt containers near heat. This study identified that out of the total study respondents, more than half had good practices of iodized salt utilization, 178 (58.3%) [95% CI (42.8–60.6)] (Table 5).

**Table 5:** Practice of the utilization of iodized salt by pregnant women in selected health centers of Addis Ababa, Ethiopia, 2022.

**Variables**	**Category**	**Frequency (*n =* 463)**	**Percentage (%)**
Types of salt buy and use	Regular	33	7.1
	Iodized	31	6.7
	Mixed	399	86.2
Place where purchase salt	Mini shop	409	88.3
	Consumer association	40	8.6
	Open market	14	3
Type of container used to store at home	Container with lid	381	82.3
	Container without lid	29	6.3
	Polyethylene	53	11.4
Expose the salt to sunlight	Yes	50	10.8
	No	413	89.2
Place where the iodized salt container is stored	Near heat	237	51.2
	Far away from heat	226	48.8
Wash salt before use	Yes	55	11.9
	No	408	88.1
The right time to add iodized salt to the meal	In the beginning	32	6.9
	Half way through cooking	60	13
	After cooking	172	37.1
	Toward the end	199	43
Overall practice of pregnant mother	Poor practice	193	41.7
	Good practice	270	58.3

The chi-square test indicated that there was a significant relationship between the knowledge and practice of pregnant women attending antenatal care for iodized salt utilization (Pearson chi-square = 441.887, *p*-value = 0.000).

The correlation indicated that there was a very strong positive correlation between the knowledge and practice of pregnant women attending antenatal care for iodized salt utilization (Pearson correlation = 0.991, *p*-value = 0.000). This showed that the knowledge of pregnant women regarding iodized salt increases the likelihood of practicing iodized salt utilization.

### Factors associated with knowledge of pregnant women about iodized salt utilization

This study found that educational status, occupational status, monthly income, previous abortion/stillbirth, and number of pregnancies were independently associated with knowledge of iodized salt utilization. The use of iodized salt was significantly associated with the educational status of pregnant women. Pregnant women with an educational status of diploma and above had 2.5-fold odds of having good knowledge of iodized salt utilization than their counterparts (AOR = 2.580; 95% CI [0.128–0.522]). Pregnant women whose occupational status was non-government workers and self-employed had 3- and 5-fold odds of having good knowledge about iodized salt utilization than their counterparts, respectively (AOR = 3.25; 95% CI [1.546–6.829]) and (AOR = 5.615; 95% CI [2.446–12.887]). Pregnant women with a previous history of abortion/stillbirth had 47% lower odds of having good knowledge of iodized salt utilization than pregnant women with no previous history of abortion/stillbirth (AOR = 0.531; 95% CI [0.319–0.882]) (Table 6).

**Table 6:** Factors associated with knowledge of pregnant women about iodized salt utilization in selected public health facilities of Addis Ababa, Ethiopia, 2022 (*n* = 463).

**Variables**	**Knowledge**				**COR (95% Cl)**	**AOR (95% Cl)**	***P*-value**
	**Good**		**Poor**				
	**No**	**%**	**No**	**%**			
**Age**							
15–24 (Ref)	7	2	15	3	1	1	
25–34	75	16	90	19	0.565 [0.202–1.579]	0.513 [0.181–1.451]	0.208
35–44	100	22	103	22	0.636 [0.326–1.242]	0.286 [0.097–0.846]	0.204
45–49	21	5	52	11	0.597 [0.311–1.148]	0.409 [0.117–1.430]	0.162
**Educational status**							
No formal education (Ref)	30	16	36	8	1	1	
Primary education	57	12	74	16	0.911 [0.509–1.634]	0.558 [0.269–1.156]	0.116
Secondary education	57	12	55	12	1.426 [0.783–2.598]	0.561 [0.281–1.122]	0.102
Diploma and above	59	13	95	21	2.071 [1.157–3.708]	2.580 [0.128–0.522]^*^	0.002
**Occupational status**							
Government	54	12	58	13	1.484 [0.920–2.392]	1.252 [0.635–2.467]	0.516
Non-government	38	8	35	8	1.099 [0.624–1.936]	3.250[1.546–6.829]^*^	0.002
Self-employed	20	4	39	8	0.547 [0.303- 0.987]	5.615[2.446–12.887]^*^	0.001
Housewife (Ref)	91	20	128	28	1	1	
**Monthly income**							
< 500 (Ref)	19	4	6	1	1	1	
500–1,500	38	8	22	5	0.991 [0.373–2.632]	2.033 [0.663–6.233]	0.215
Above 1,500	112	24	176	38	0.716 [0.306–1.673]	2.731 [1.130–6.600]^*^	0.026
Unknown	34	7	56	12	0.771 [0.306,1.947]	0.682 [0.372–1.253]	0.218
**Pervious abortion/stillbirth**							
Yes	148	32	160	35	0.993 [0.668–1.475]	0.531 [0.319–0.882]	0.014
No (Ref)	55	12	100	22	1	1	
**Number of ANC visit**							
1	16	16	16	3	1	1	
2–3	64	14	94	20	1.556 [0.651–3.719]	0.162 [0.054–0.481]	0.001
≥4	123	27	150	32	2.288 [0.981–5.340]	0.598 [0.366–0.979]	0.041
**Number of pregnancies**							
One (Ref)	98	21	78	17	1	1	
Two	55	12	72	16	0.918 [0.580–1.452]	1.482 [0.866–2.537]	0.151
Three	37	8	62	13	2.097 [1.235–3.560]	5.403 [2.653–11.001]^*^	0.001
> Four	13	3	48	10	2.753 [1.380–5.490]	5.656 [2.398–13.341]^*^	0.001
**Receive information during ANC**							
Yes	15	3	47	10	1.295 [0.753–2.228]	0.701 [0.373–1.319]	0.271
No (Ref)	188	41	213	46	1	1	

^*^Significantly associated variables at *p*-value < 0.05, 1-Reference group. AOR, Adjusted odds ratio; COR, crude odds ratio.

Pregnant women with 2–3 and ≥4 ANC visits had 84% and 40% lower odds of having good knowledge of iodized salt utilization than their counterparts (AOR = 0.16; 95% CI [0.054–0.461] and AOR = 0.598; 95% CI [0.366–0.979], respectively). Women who had a monthly income above 1,500 ETB were 2.7 times (AOR = 2.731; 95% CI: 1.13–6.6) more likely to have good knowledge than women who did not. Having three or more children was also significantly associated with good knowledge of iodized salt utilization (AOR = 5.4; 95% CI [2.65–11.00]) (AOR = 5.65; 95% CI [2.39–13.34]) (Table 6).

### Factors associated with the practice of pregnant women about iodized salt utilization

In this study, diploma and above educational status, having a self-employed occupation, the number of ANC visits, and having three, four, and above pregnancies were significantly associated with iodized salt utilization practices (*p* < 0.05). Having diplomas and above was 4.63 times more likely to have good iodized salt utilization practice when compared to no formal education [AOR = 4.63; 95% CI: (2.035–10.537)]. Respondents with self-employed occupational status were 73% less likely to have good iodized salt utilization practices [AOR = 0.275; 95% CI: (0.137–0.552)] than those who were housewives. The odds of good iodized salt utilization were higher among those who had four or more ANC visits [AOR = 6.301, 95% CI: (2.110–18.815)] as compared to those who had one ANC visit. Respondents with three, four, and more pregnancies were found to have 78% and 10% lower odds of having good iodized salt utilization practice [AOR = 0.226; 95% CI: (0.097–0.525) and AOR = 0.906; 95% CI: (1.379–2.165), respectively] compared to those who had one pregnancy (Table 7).

**Table 7:** Factors associated with practice of iodized salt utilization in pregnant women in selected public health facilities of Addis Ababa, Ethiopia, 2022 (*n* = 463).

**Variables**	**Practice**				**COR (95% Cl)**	**AOR (95% Cl)**	***P*-value**
	**Good**		**Poor**				
	**No**	**%**	**No**	**%**			
**Age (years)**							
15–24 (Ref)	11	2	11	2	1	1	
25–34	82	18	83	18	0.565 [0.202–1.579]	2.636 [0.748–9.282]	0.276
35–44	108	23	95	21	0.636 [0.326–1.242]	1.269 [0.556–2.898]	0.185
45–49	21	5	52	11	0.564 [0.293–1.083]	0.651 [0.305–1.389]	0.085
**Educational status**							
No formal education (Ref)	35	16	31	7	1	1	1
Primary education	66	14	65	14	0.911 [0.509–1.634]	1.261 [0.637–2.498]	0.756
Secondary education	57	12	55	12	1.426 [0.783- 2.598]	3.247 [1.541–6.840]	0.246
Diploma and above	64	14	90	19	1.885 [1.056–3.363]	4.630 [2.035–10.537]^*^	0.032
**Occupational status**							
Government	57	12	55	12	1.484 [0.920–2.392]	0.644 [0.313–1.324]	0.105
Non-government	45	10	28	6	0.908 [0.518–1.589]	0.453 [0.226–0.906]	0.734
Self-employed	18	4	41	9	0.547 [0.303–0.987]	0.275 [0.137–0.552]^*^	0.045
Housewife (Ref)	102	22	117	25	1	1	1
**Monthly income**							
< 500 (Ref)	17	4	8	2	1	1	1
500–1,500	44	10	16	3	1.296 [0.514–3.271]	2.136 [0.695–6.564]	0.583
Above 1,500	127	27	161	35	1.285 [0.644–2.562]	2.913 [1.200–7.070]	0.177
Unknown	34	7	56	12	0.889 [0.542–1.457]	0.697 [0.380–1.280]	0.24
**Pervious abortion/stillbirth**							
Yes	149	32	159	34	1.050 [0.707–1.558]	0.531 [0.320–0.882]	0.21
No (Ref)	73	16	82	18	1	1	1
**Number of ANC visit**							
1 (Ref)	19	16	13	3	1	1	1
2–3	59	13	99	21	1.437 [0.601–3.436]	3.477[1.203–10.049]	0.415
> Four	144	31	129	28	2.288 [0.981–5.340]	6.301 [2.110–18.815]^*^	0.056
**Number of pregnancies**							
One (Ref)	97	21	79	17	1	1	1
Two	62	13	65	14	0.830 [0.524–1.314]	0.166 [0.071–0.389]	0.427
Three	40	9	59	13	2.097 [1.235–3.560]	0.226 [0.097–0.525]	0.006
>Four	23	5	38	8	2.753 [1.380–5.490]	0.906 [1.379–2.165]	0.004
**Receive information during ANC**							
Yes	15	3	47	10	0.937 [0.550–1.597]	0.865 [0.461–1.620]	0.12
No (Ref)	207	45	194	42	1	1	1

^*^Significance at *p*-value < 0.05, 1- Reference group. AOR, Adjusted odds ratio; COR, Crude odds ratio.

## Discussion

This study aimed to investigate the knowledge, practices, and factors associated with iodized salt use among ANC-tending women in public facilities in Addis Ababa, Ethiopia. Based on this study, 41.1% of women attending ANC had good knowledge of iodized salt utilization. This finding was higher than those reported in Gondar town (25.2%) (27), Mercha district (28.7%) (24), and Addis Ababa (27%) (17). This might be because mothers living in urban areas and capital cities have better education and more chances of acquiring nutrition and other health-related information from different sources (17).

However, the results of this study were lower than those of other studies conducted in East Welega, Ethiopia (64.4%) (28), and Ghana (90.4%) (4). This difference might be due to sociodemographic differences and the high attention paid to counseling about nutrition during practices of women attending ANC visits in Welega, Ethiopia, and a special focus of the Ghanaian government toward improving maternal and child health problems related to iodine deficiency in the country (29).

In this study, 58.3% of the women who attended ANC had good iodized salt utilization practices. This finding was slightly higher than that of a study conducted in Gondar Town (40.1%) (30), Northwest Ethiopia (39.3%) (22), and Dessie Town (54.8%) (31), which reported good dietary practices among pregnant women. A possible reason for the difference between these study findings may be that pregnant women living in big cities might have more access to health education about iodized salt utilization, which may account for better practices of handling iodized salt than in small cities and districts (17). The iodized salt utilization practices of the study participants were correspondingly higher than those in Welega Zone, Ethiopia (25.1%). This might be due to differences in the respondents' educational status (28). However, similar to a study conducted among pregnant mothers in the Kimbitibit District North Shoa Zone, which indicated that 48.1% and 45.4% of respondents in Jibat Woreda, West Shoa Zone, Ethiopia had good iodized salt utilization (13, 32). This finding was lower than that in the study done in Addis Ababa City, which showed that 76.3% of households had good practices regarding iodized salt, but higher than the study done in Tehran, which showed that 14% of households had good practices regarding iodized salt (33, 34).

Regarding educational status, study participants with secondary education and a diploma and above had higher odds of practicing good iodized salt utilization during pregnancy than those who were not. This is similar to the findings from southwestern Bangladesh (35), the Gedeo zone (36), Jibat (32), Wolayita (37), and Gondar town (30). A possible explanation for this is that mothers with better education have a higher chance of acquiring nutrition and health-related information from different sources such as leaflets, magazines, and other media (24, 38) and possess better reading and comprehension skills, enabling them to understand and implement health practices more effectively than those who are unable to read and write (39).

The occupational status of the participants was one of the factors associated with knowledge of iodized salt use among women attending the ANC. Pregnant women whose occupational status was non-governmental and self-employed were more likely to have good knowledge of iodized salt utilization. This finding is also supported by a study conducted in the Mecha District. The reason for this might be that the educated heads of households have learned and read about the importance of iodized salt (24). Conversely, a study conducted in Ambo (25) indicated that the government and farmers were 11.1 times 95% CI (1.33–92.91) and 13.86 times (1.64–117.24) more likely to have been knowledgeable than their counterparts, respectively.

The present study revealed an association between iodized salt use and number of pregnancies. Having three or more children was significantly associated with good knowledge of iodized salt utilization. This finding was consistent with a study conducted in public hospitals in Addis Ababa, given that the number of pregnancies was positively associated with good nutrition practices during pregnancy (17).

Households with a monthly income of >1,500 ETB increased the odds of good knowledge of iodized salt utilization by 2.73, which were factors associated with the availability of iodized salt. A study conducted in Ghana (3) revealed that, compared to the richest category, all other lower levels of wealth were more likely to use iodized salt. It also shows that wealth is a significant determinant of the likelihood of adequate iodized salt use (40). A similar study conducted in Pakistan reported that income plays an important role and is the most important determinant in achieving adequate nutrition in the household (41). This finding is also supported by a study conducted in Southern Ethiopia, Sidama Zone (42), Bensa Woreda, and Asella Town, Arsi Zone (43). This finding was consistent with a study conducted in the Gedeo zone, which also revealed monthly income as a significant predictor of good iodized salt utilization practices. A possible explanation for this is that self-reliant mothers can afford different food items and feed the whole family as well as themselves. A similar finding was reported from a study conducted in northwestern Ethiopia Gonder (30) town, which reported that respondents who earn between 28.5 and 57 dollars/month have two times more odds of having good dietary practices than those earning 57 dollars and those who earn between 28.5 and 57 dollars were 3.17 and 2.84 times more odds of practicing good diet than those whose monthly income of earning was below 28.5 dollars.

The other predictor variable for iodized salt utilization was ANC visits. This study revealed that women who had ANC follow-ups were more likely to have good iodized salt utilization practices than those who did not. This finding is consistent with a study conducted in the Gedeo zone, which showed that mothers who had no ANC follow-up were 54% less likely to have good dietary practices than mothers with ANC follow-up (36).

Iodized salt use in households might not be a good indicator of sufficient daily iodine intake. It can be lost owing to different types of cooking practices, such as pressure cooking, boiling, deep-frying, and shallow frying. This is one of the improper uses of iodized salt in pregnant women and the general community (44).

## Strengths and limitation of the study

This study used large sample sizes compared to previous studies, which decreased the sampling error.

The study design was cross-sectional; therefore, it did not reveal a causal relationship.

This study was specific to government health facilities.

The study would be better representative of the whole community if it was a community-based study instead of an institution-based study.

## Conclusion

Based on the findings of the present study, it can be concluded that women attending ANC had low levels of iodized salt knowledge and practice in the study area. There was a significant positive relationship between the level of education, monthly income, occupational status, ANC visits, and number of pregnancies with iodized salt utilization knowledge and practices. Hence, nutrition interventions such as nutrition education in different villages, health centers, health institutions, and women's organizations should be given to the community, particularly for pregnant mothers concerning nutrition during pregnancy, to increase the knowledge and practice of the utilization of iodized salt.

## Recommendations

### Sub-cities (Yeka, Lemi Kura, and Nefassilk Lafto)

All health facilities providing nutritional services, specifically for pregnant women, need continuous monitoring and evaluation to maintain service quality.

### Addis Ababa City Administration health bureau

Capacity-building activities for health professionals should be provided for iodine salt utilization and its value in pregnant women.

Strengthen the monitoring and evaluation of nutrition services for pregnant women in government health facilities.

### Policymakers

Must consider nutrition services and proper counseling specifically for pregnant women at the grass-route level, specifically for primary health facilities, including governmental health centers.

The monitoring and evaluation strategy for overall nutrition service for pregnant women is markedly incorporated in policy documents to sustain the coverage and quality of nutrition service at all levels.

### Researchers

Further investigation should be conducted using qualitative studies to identify the causes of low iodine salt utilization.

## Data Availability

The raw data supporting the conclusions of this article will be made available by the authors, without undue reservation.
