# Supplementary material for: Knowledge and practice of iodine salt utilization and associated factors among pregnant women attending antenatal care in public health facilities in Addis Ababa, Ethiopia
Source: Front Nutr. 2025 Jun 19;12:1529842. doi: 10.3389/fnut.2025.1529842 (PMC12239141; doi:10.3389/fnut.2025.1529842)
Supplement: Supplementary file 1 [file Table_1.docx]

ANNEX

Annex 1: English Version of Participant’s Consent and Information Sheet

| Date of data collection _____ / _____ / ______ (Ethiopian calendar: Day Month Year)  Sub city ---------------Woreda……….. Questionnaire code ______  Name of data collector__________________________ Sign__________________  Name of supervisor_____________________________ Sign __________________  Unique Identification Number ……………………..  Circle only one answer | | |
| --- | --- | --- |
| Part One: SOCIO DEMOGRAPHIC INFORMATION | | **No Question Response Skip pattern** |
| 101 | How old are you? Age in years completed at the last birthday | --------------------------------------- |
| 102 | Religion | 0.Orthodox  1. Protestant  2. Muslim  3. Catholic  4. Other Specify |
| 103 | What is your occupational status? | 0. Government employee  1. Non-government employee  2. Self-employed  3. House wife |
| 104 | What is your marital status? | 0. Married  1. Single-never married  2. Widowed  3. Divorced |
| 105 | What is the highest level of education you have completed? | 0.No formal education  1. Primary Education (1-8)  2. Secondary Education(9-12)  3.College Diploma and above |
| 106 | How many people are there in your household? ( family size) | _______Number of People |
| 107 | Monthly income | 0.Less than 500 ETB  1. 500-1500ETB  2. More than 1500ETB  3.Not known |
|  | PART TWO: REPRODUCTIVE AND ANC FOLLOW UP RELATED CHARACTERSTICS OF WOMEN | |
| 201 | How many pregnancies did you have before? | 0.One  1.Two  2.Three  3.Four and above |
| 202 | Is there abortion or still birth? | 0.No if No Skip to Q#204  1. Yes |
| 203 | If yes, what was the reason? | 0. RH incompatibility  1. Accident  2.Fetal distress  3.Bleeding  4.Nutration  5.I Don’t Know |
| 204 | How many times to here for ANC Visit | 1. 1 2. 2-3 3. > 4 |
| 205 | Who was your counselor for the last ANC visit? | 1. HO/BSc 2. GP 3. Midwife |
| 206 | Did you received information/health education during ANC follow up? | 1. No If No skip to Question 301 2. Yes |
| 207 | What information given during ANC follow up? | 1. Hygiene and sanitation 2. Nutrition 3. Danger sign 4. Birth preparedness 5. All |
| 208 | If you discussed about nutrition ,give information about iodine and iodized salt utilization | 1. No 2. Yes |
|  | PART THREE: QUESTIONS RELATED TO KNOWLEDGE OF IODINE AND IODIZED SALTS. | |
| 301 | Have you ever heard about iodine? | 0. No 1. Yes |
| 302 | Have you heard about the problems of a deficiency of iodine in environment and insufficient intake of iodine in the human body? | 0. No 1. Yes |
| 303 | Have you ever heard of the effects iodine deficiency on the human body? | 0. No 1. Yes |
| 304 | Do you think iodine deficiency problems exist in Ethiopia? | 0. No 1. Yes ,If your answer is No skip to question “210” |
| 305 | Which problems (disorders) of IDDs are you heard? | 1. Still birth 2. Heart Pain 3. Retarded development 4. Reduced immunity 5. Memory lose 6. Goiter |
| 306 | How can we prevent IDDS? | 1. Physical activity 2. Using Iodized salts 3. Making tattoo 4. Other 5. I don’t know |
| 307 | Have you ever heard of about iodized salt? | 1. No 1. Yes |
| 308 | If yes to (305) where did you hear of it? | 1. Printed materials 2. Television/Radio 3. Health workers 4. Family 5. School 6. Other |
| 309 | Can you mention types of salts available in the market? | 1. Regular 2. Iodized 3. Mixed |
| 308 | Does every salt contains iodine? | 1. No 2. Yes |
| 309 | Is using iodized salt important for human health? | 1. No 2. Yes |
| 310 | Iodine is important for? | 1. prevent blindness 2. Normal fetal development 3. Strengthen teeth and skeleton 4. Maintain normal metabolism 5. Prevent spina bufida 6. I don’t know |
| 311 | What are the most important dietary iodine sources other than iodized salt? | 1. Fish 2. Meat 3. Vegetable oil 4. Dairy Product 5. I Don’t know |
| 312 | What are the advantage of iodized salt over non-iodized? | 1. Better taste 2. Easily soluble 3. Replace iodine deficiency 4. I Don’t know |
| 313 | Do you think if the pregnant woman has lack of iodine which makes the fetus birth before EDD? | 1. No 2. Yes 3. I Do not know |
| 314 | Do you think iodine deficiency will make children learn below the normal? | 1. No 2. Yes 3. I Do not know |
| 315 | Do you agree the family suffer by goiter with using cooking Iodized salt for so many years? | 1. No 2. Yes 3. I Do not know |
| 316 | Do you think you Can see and differentiate b/n non Iodized salt and Iodized salt? | 1. No 2. Yes 3. I Do not know |
| 317. | Why do you buy iodized salt? | 1. I know that it is healthy 2. Other salt are not available 3. other 4. I Don’t know |
| 318. | Should salt packaging have information for the buyer about the contents of the product? | 1. No 2. Yes 3. I don’t know |
| 319. | What information would you like to see on the packaging for iodized salt? | 1. Weight 2. Contents 3. Health values 4. Period of storage 5. Producer 6. More than two answer |
| 320. | Where Do you kept iodized salt container? | 1. Near heat 2. Far away from heat and sunlight |
| 321 | Is the test of iodized salt is different from that of common salt? | 1. No 2. Yes 3. I Don’t Know |
|  | PART FOUR- PRACTICE OF IODIZED SAT |  |
| 401 | Which types of salt buy and use? | 1. Regular 2. Iodized 3. Mixed |
| 402 | If your answer is regular, why don’t buy and use iodized salt? | 1. I Don’t Know why it is necessary 2. It is usually More expensive 3. Member of May family advice 4. My friend Advise me |
| 403 | If your answer is iodized, why do you buy iodized salt? | 1. I Know it is health 2. Other kinds of salts not available 3. Information given from health professional 4. The sales person inform me |
| 404 | Where you usually purchased salt? | 1. Mini-shop 2. consumer association 3. open market 4. other |
| 405 | What type of container used to store salt at home? | 0.Container with lid  1.Container without lid  2.Polyethylene bag |
| 406 | At what time should salt be added while cooking | 0.In the beginning  1.Halfway through cooking  2.After cooking  3.Towards the end |
| 407 | Do you expose the salt to sun light? | 1. No 2. Yes |
| 408 | Do you wash salt before use? | 1. No 2. Yes |
| 409 | Does the presence of iodine in a salt affect a test? | 1. No 2. Yes |

## **Annex 2: Amharic Version of Participant’s Consent and Information Sheet**

| መረጃየተሰበሰበትቀን ----------------------/----------/(በኢትዮጲያ አቆጣጠር)  ከ/ከተማ ወረዳ የመጠይቅ ቁጥር  የመረጃ ሰብሰቢዉ ስም ፊርማ  የአስተባባሪዉ ስም ፊርማ  መልሱን ብቻ ያክብቡ  ስለማህበራዊ እና ኢኮኖሚ ሁኔታን በተመለተከተ  መለያኮድ | | | | | | | | |
| --- | --- | --- | --- | --- | --- | --- | --- | --- |
| 1. | ጾታ | 1. ወንድ 2. ሴት |  | | | | |  |
| 2. | እድሜሽስንትነዉ? |  |  | | | | |  |
|  | ሃይማኖት | 1. ፕሮቴስታንት 2. ኦርቶዶክስ 3. ሙስሊም 4. ካቶሊክ 5. ሌላካለጥቀሺ |  | | | | |  |
| 4. | የስራ/ሽሁኔታ | 1. የመንግስትሰራትኛ፤መንግስታዊያልሆነ 2. የቀንሰራተኛ 3. የግልሰራተኛ 4. ገቢየሌላዉ |  | | | | |  |
|  | የጋብቻ ሁኔታ | 1. ያላገባ 2. ያገባ 3. የትዳር አጋር የሞተበት 4. የተፋታ 5. የተለያየ |  | | | | |  |
| 6. | የትምህርት ደረጃ | 1. የመጀመርያ ደረጃ 2. ሁለተኛ ደረጃ 3. ዲፕሎማ 4. ድግሪ 5. ከዛበ ላይ |  | | | | |  |
| 7. | በቤትሽ ዉስጥ ምን ያህል ቤተሰብ አለ? | የሰዉብዛት |  | | | | |  |
| 8. | የወር ገቢ | 1.500 ብር በታች  2. 500-1500 ብር  3. 1500 ብር በላይ  4.አላዉቀዉም |  | | | | |  |
| 9. | ከዚህ በፊት ስንት ግዜ ጸንሰሻል? |  |  | | | | |  |
| ስለአዮዲን እዉቀት ወይም ግንዛቤ በተመለከተ | | |  |  |  |  |  |  |
| 10. | አዮዲንምንእንደሆነታዉቂያለሽ? | | 1. አዎ   0.አላዉቅም | |  | |  |  |
| 11. | ስለአዮዲንሰምተሸታዊቂያለሽ? | | 1. አዎ   0.አላዉቅም  2. አላስታዉስም | |  | |  |  |
| 12. | መረጃዉንከየትአገኝሽ? | | 1.ከመፅሔት  2.ከቴሌቪዝን  3. ከጤናባለሙያ  4.ከቤተሰብ  5.በት/ቤት  6.ሌላካለ | |  | |  |  |
| 13. | አዮዲንለምንይጠቅማል? | | 1. ለህጻንልጅእድገትናጥንካሬ 2. አይነስዉርነትንለመከላከል 3. ለፅንስእድገትናመዳበር 4. ለጥርስናለአጥንትጥንካሬ 5. በሰዉነትዉስጥሜታቦሊዝምንለማስተካከል 6. እሰፒንባፊዳንለመከላከል 7. አላዉቀዉም | |  | |  |  |
| 14. | በጣምጠቃሚየአዮዲንምንጭምንድንነዉ? | | 1. አሳ 2. የአትክልትወተት 3. ስጋ 4. የአትክልትዘይት 5. ዳቦናየወተትተዋፆ 6. አላዉቀዉም | |  | |  |  |
| 15. | በአዮዲንየበለፀገጨዉለሰዉልጅጤናይጠቅማል? | | 1አዎ  0.አላዉቅም  2. አላስታዉስም | |  | |  |  |
| 16 | የአዮዲንእጥረትጋርተያይዞያለየጤናችግርበኢትዮጲያአለ? | | 1አዎ  0.አላዉቅም  2. አላስታዉስም | |  | |  |  |
| 17. | እንቅርትበአይንይታያል? | | 1አዎ  0.አላዉቅም  2. አላስታዉስም | |  | |  |  |
| 18. | የአዮዲንእጥረትምክንያትየሚከሰቱየጤናችግሮችእነማንናቸዉ? | | 1. እንቅርት 2. የአይምሮህመም 3. ጽንስመጨናገፍ 4. የጨቅላህጻንሞት 5. ሌላካለ | |  | |  |  |
| 19. | የአዮዲንእጥረትምክንያትየሚከሰቱየጤናችግሮችእንዴትመከላከልእንችላለን? | | 1.የሰዉነትእንቅስቃሴበመስራት  2. አዮዲንየበለፀገጨዉበመጠቀም  3.በመነቀስ  4. ሌላካለ | |  | |  |  |
| 20. | አንድነፍሰጡርእናትበአዮዲንእጥረትምክንያትከቀኗቀድማልትወልድትችላለች? | | 1አዎ  0.አላዉቅም  2. አላስታዉስም | |  | |  |  |
| 21. | የአዮዲንእጥረትበሕጻናትእድገትላይተፅኖአለዉ? | | 1አዎ  0.አላዉቅም  2. አላስታዉስም | |  | |  |  |
| 22. | አንድሰዉአዮድንየተመጣጠነምግብበመብላቱከእንቅርትለብዙዓመትሊከላከልይችላል? | | 1አዎ  0.አላዉቅም  2. አላስታዉስም | |  | |  |  |
| 23. | አዮዳይዝድጨዉንካልሆነዉመለየትይቻላል? | | 1አዎ  0.አላዉቅም  2. አላስታዉስም | |  | |  |  |
| 24. | አዮዳይዝድጨዉለምንድንዉገዝተንየምንጠቀመዉ? | | 1.ጤናማእንደሆነስለማዉቅ  2.ሌላአይነትጨዉስለማይገኝ  3. ሙያዊምክርስላገኝዉ  4. | |  | |  |  |
| 25. | አዮዲዝድጨዉእለትተእለትመጠቀማችንያለዉንጠቀሜታታዉቂያለሽ? | | 1አዎ  0.አላዉቅም | |  | |  |  |
| 26. | በጨዉመጠቅለያላይአስፈላጊመረጃዎችመኖርአለባቸዉብለሽታምኛለሽ? | | 1አዎ  0.አላዉቅም  2. አላስታዉስም | |  | |  |  |
| 27. | የጨዉማሸጊያላይምንምንመኖርአለበት? | | 1. ክብደት 2. ይዘት 3. የጤናጥቅም 4. ለምንያሃልግዤእንደሚያገለግል 5. አምራች 6. ሌላካለ | |  | |  |  |
| 28. | አዮዳይዝጨዉእሳትአጠገብቢቀመጥችግርአለዉ? | | 1አዎ  0.አላዉቅም  2. አላስታዉስም | |  | |  |  |
| የአዮዳዝድጨዉአጠቃቀምዝንባሌበተመለከተ | | |  |  |  |  |  |  |
| 31. | የባህርወይምየአሳምግቦችንበየስንትግዜትመገባላችሁ? | | 1.በየቀኑ  2.በየሳምንቱ  3.በወርአንዴ   1. አልፎአልፎ 2. ከረጂምጊዜበዉኃላ 3. በፍፁምአልበላም |  | |  |  |  |
| 32. | አዮዳዝድጨዉተጠቅመሽታቂያለሽ? | | 1.አዎ  0.አላዉቅም |  | |  |  |  |
| 33 | ለምንተጠቅመሽአታዉቂም? | | 1. ጠቃሚእንደሆነአላዉቅም 2. ዋጋዉዉድስለሆነ 3. ቤተሰቦቼተጠቅመዉአያዉቁም 4. ስለጠቀሜታዉሰምቼአላዉቅም |  | |  |  |  |
| 34. | አዮዳይድጨዉለምንደንነዉየምንትጠቀሚዉ? | | 1.ጤናማእንደሚያደርገኝስለማዉቅ  2.ሌላጨዉስለሌለ  3.ከሃኪምምክርስላገኝዉ  4.ከሻጩምክርስላገኘዉ  5.በቤተሰብግፊት  6. በጓደኞቼምክንያት  7. በስራባልደረቦቼግፊት  8.በጎረቤቶቼተፅኖ |  | |  |  |  |
| 35. | በቤትዉስጥጨዉእንዴትነዉየምታስቀምጪዉ? | | 1.በተሸፈነጣሳዉስጥ  2.ባልተሸፈነጣሳዉስጥ  3.ፖሊኢታይሊንቀረጢት |  | |  |  |  |
| 36. | አዮዲንበጨዉዉስጥመኖሩየጣዕምለዉጥያመጣል? | | 1.አዎ  0.የለዉም |  | |  |  |  |
| 37. | ጨዉከየትነዉየምታገኚዉ? | | 1.ከሱቅ  2.ከባዛር  3.ሌላ |  | |  |  |  |
| 38 | የታሸገበትበጠቅለያስለግብአቶችመዘርዝርአለዉ? | | 1.አዎ  0.የለዉም |  | |  |  |  |
| 39. | በማሸጊያዉላይአዮዳይዝጨዉየሚልስያሜአለዉ | | 1.አለ  2.የለም |  | |  |  |  |
| 40. | ሎጎአለ? | | 1.አለ  2.የለም |  | |  |  |  |
